# The effect of blood pressure on mortality following out-of-hospital cardiac arrest: a retrospective cohort study of the United Kingdom Intensive Care National Audit and Research Centre database

**DOI:** 10.1186/s13054-022-04289-2

**Published:** 2023-01-05

**Authors:** Peter J. McGuigan, Elisa Giallongo, Bronagh Blackwood, James Doidge, David A. Harrison, Alistair D. Nichol, Kathryn M. Rowan, Manu Shankar-Hari, Markus B. Skrifvars, Karen Thomas, Danny F. McAuley

**Affiliations:** 1 Regional Intensive Care Unit, Royal Victoria Hospital, Belfast, UK; 2 Wellcome-Wolfson Institute for Experimental Medicine, Queen’s University, Belfast, UK; 3 Intensive Care National Audit and Research Centre, Napier House, 24 High Holborn, London, UK; 4 University College Dublin Clinical Research Centre, St Vincent’s University Hospital, Dublin, Ireland; 5 The Australian and New Zealand Intensive Care Research Centre, Monash University, Melbourne, Australia; 6 The Alfred Hospital, Melbourne, Australia; 7 Centre for Inflammation Research, Institute of Regeneration and Repair, University of Edinburgh, Edinburgh, UK; 8 Royal Infirmary of Edinburgh, NHS Lothian, Edinburgh, UK; 9 Department of Emergency Care and Services, University of Helsinki, Helsinki, Finland; 10 Helsinki University Hospital, Helsinki, Finland

**Keywords:** Cardiac arrest, Blood pressure, Mean arterial pressure, Systolic blood pressure, Hypotension, Hypertension, Critical care, Mortality

## Abstract

**Background:**

Hypotension following out-of-hospital cardiac arrest (OHCA) may cause secondary brain injury and increase mortality rates. Current guidelines recommend avoiding hypotension. However, the optimal blood pressure following OHCA is unknown. We hypothesised that exposure to hypotension and hypertension in the first 24 h in ICU would be associated with mortality following OHCA.

**Methods:**

We conducted a retrospective analysis of OHCA patients included in the Intensive Care National Audit and Research Centre Case Mix Programme from 1 January 2010 to 31 December 2019. Restricted cubic splines were created following adjustment for important prognostic variables. We report the adjusted odds ratio for associations between lowest and highest mean arterial pressure (MAP) and systolic blood pressure (SBP) in the first 24 h of ICU care and hospital mortality.

**Results:**

A total of 32,349 patients were included in the analysis. Hospital mortality was 56.2%. The median lowest and highest MAP and SBP were similar in survivors and non-survivors. Both hypotension and hypertension were associated with increased mortality. Patients who had a lowest recorded MAP in the range 60–63 mmHg had the lowest associated mortality. Patients who had a highest recorded MAP in the range 95–104 mmHg had the lowest associated mortality. The association between SBP and mortality followed a similar pattern to MAP.

**Conclusions:**

We found an association between hypotension and hypertension in the first 24 h in ICU and mortality following OHCA. The inability to distinguish between the median blood pressure of survivors and non-survivors indicates the need for research into individualised blood pressure targets for survivors following OHCA.

**Graphical Abstract:**

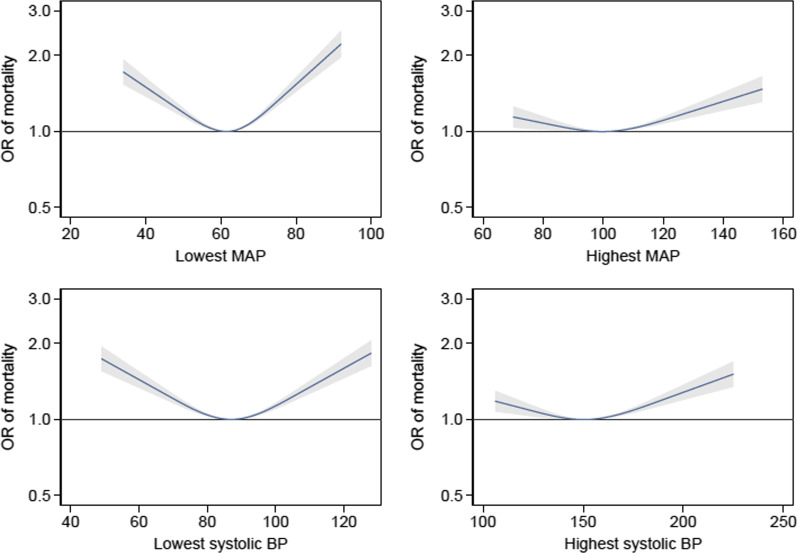

**Supplementary Information:**

The online version contains supplementary material available at 10.1186/s13054-022-04289-2.

## Introduction

Each year in the UK, 3800 patients require mechanical ventilation in ICU following an out-of-hospital cardiac arrest (OHCA) [1]. Unfortunately, ICU and hospital mortality for these patients remains persistently high with hypoxic ischaemic encephalopathy responsible for the majority of deaths [2, 3]. Survivors have significant physical, cognitive, and emotional sequelae.^4^

Hypoxic ischaemic encephalopathy following OHCA may result in loss of or “rightward shift” in cerebral autoregulation, rendering cerebral blood flow dependent on cerebral perfusion pressure (CPP) [5]. Hypotension may exacerbate cerebral ischaemia, causing secondary brain injury and increase mortality [6, 7]. Blood pressure management strategies in ICU represent one deliverable therapeutic option which may improve mortality for patients following OHCA [8].

A number of observational studies have demonstrated an association between hypotension and mortality following OHCA [4, 9–20]. Current guidelines recommend avoiding hypotension (defined as a MAP < 65 mmHg or a systolic blood pressure (SBP) of < 90 mmHg) [4, 8]. However, the risk associated with exposure to hypertension is poorly described [21]. The optimal blood pressure target for patients following OHCA remains unclear [8]. The European Resuscitation Council has called for “research into identification of optimal MAP targets for individual cardiac arrest survivors receiving intensive care” [4]. Thus, an analysis of a large multicentre registry is needed to identify blood pressure targets that could be tested as an intervention in a randomised controlled trial.

We hypothesised that exposure to hypotension and hypertension in the first 24 h in ICU would be associated with mortality following OHCA.

## Materials and methods

All intensive care units in England, Wales, and Northern Ireland contribute to the Intensive Care National Audit & Research Centre (ICNARC) Case Mix Programme (CMP), a national comparative audit of patient outcomes [22]. Trained data collectors collect data on physiological parameters and non-physiological predictors of mortality for consecutive admissions to ICU. Data undergo validation prior to pooling in the CMP. Support for the collection and use of CMP data has been obtained under section 251 of the National Health Service Act 2006 (approval number: PIAG 2-10(f)/2005).

The physiological parameters recorded include the lowest and highest SBP values, with their paired diastolic blood pressure (DBP), during the first 24 h of ICU admission. Lowest and highest MAP is calculated by applying the formula [DBP + 1/3(SBP-DBP)] to paired lowest and highest blood pressure values [18]. The ICNARC CMP does not collect serial blood pressure values over time.

A retrospective analysis of the ICNARC CMP was undertaken for the period 1 January 2010 to 31 December 2019. Cases beyond 31 December 2019 were not included as the COVID pandemic may have affected the provision of pre-hospital and intensive care services.

Adult patients aged ≥ 16 years who had suffered an OHCA were included. OHCA patients were identified in the ICNARC CMP as those admitted to ICU from “clinic or home” via the “emergency department”, a “specialist treatment area” or “imaging department” in the same hospital, and who had cardiopulmonary resuscitation (CPR) in the community in the 24 h prior to ICU admission.

We applied the following exclusion criteria; in hospital cardiac arrest (IHCA), patients who remained unintubated in the first 24 h, those admitted following trauma or surgery, readmissions to ICU within the same hospitalisation, those with missing blood pressure readings, and those with missing mortality outcomes. For the main analysis, and in keeping with previous studies [9, 10, 15, 23, 24], we excluded those who died within the first 24 h. The majority of patients who die in the first 24 h in ICU following OHCA have withdrawal of life-sustaining therapy. Perceived poor neurological outcome is the commonest reason for withdrawal of life-sustaining therapy followed by, medical instability, withdrawal for non-neurological reasons, and brainstem death [25]. Therefore, these patients’ outcomes are unlikely to be influenced by blood pressure management strategies following OHCA.

We considered MAP in the first 24 h as the primary exposure. Hospital mortality was the primary outcome measure. We report the adjusted odds ratio for associations between lowest and highest MAP and hospital mortality. The mean lowest and highest MAP values recorded were used as the reference point for mortality. The process was repeated for SBP recordings. In a series of sensitivity analyses, we repeated these analyses including those who died within the first 24 h of ICU admission.

In a regression model, restricted cubic splines were created after checking for nonlinearity to model the nonlinear relationship between blood pressure and mortality. We adjusted for the following variables: age, sex, ethnicity, pre-admission dependency, presence of severe comorbidity as defined by APACHE II, APACHE II Acute Physiology Score (excluding the MAP component as this was tested in our primary exposure), primary diagnosis category, year of OHCA, highest central temperature, and lowest glucose [3]. Further detail is provided in Additional file 1: Table S1. The adjusted analysis was conducted after multiple imputation via chained equations for missing variables. Restricted cubic splines are reported with ranges from 1st to 99th percentiles, and adjusted odds ratios for mortality are reported. In a post hoc analysis, we treated MAP and SBP as categorical variables with blood pressure divided into 12 categories ranging from MAP < 45 mmHg to ≥ 145, and SBP < 60 mmHg to ≥ 160 mmHg and report unadjusted and adjusted OR for mortality. We conducted further post hoc analyses adjusting for the use of advanced cardiovascular support. Finally, we conducted post hoc analysis using the outcome measure of mortality or failure to return to usual place of residence as a surrogate for poor neurological outcome (as neurological outcome is not recorded in the ICNARC CMP).

Baseline results are reported as mean (standard deviation), median (interquartile range) or percentage. Pearson’s chi-squared and adjusted and unadjusted odds ratios are reported for the categorical variables. P values presented are two sided, with *P* < 0.05 considered statistically significant. No adjustment was made for multiple comparisons [26]. In this retrospective cohort study, all findings were considered hypothesis generating. Analyses were performed in Stata 16.1 (StataCorp LP, College Station, TX).

## Results

### Population characteristics

**Fig. 1:**
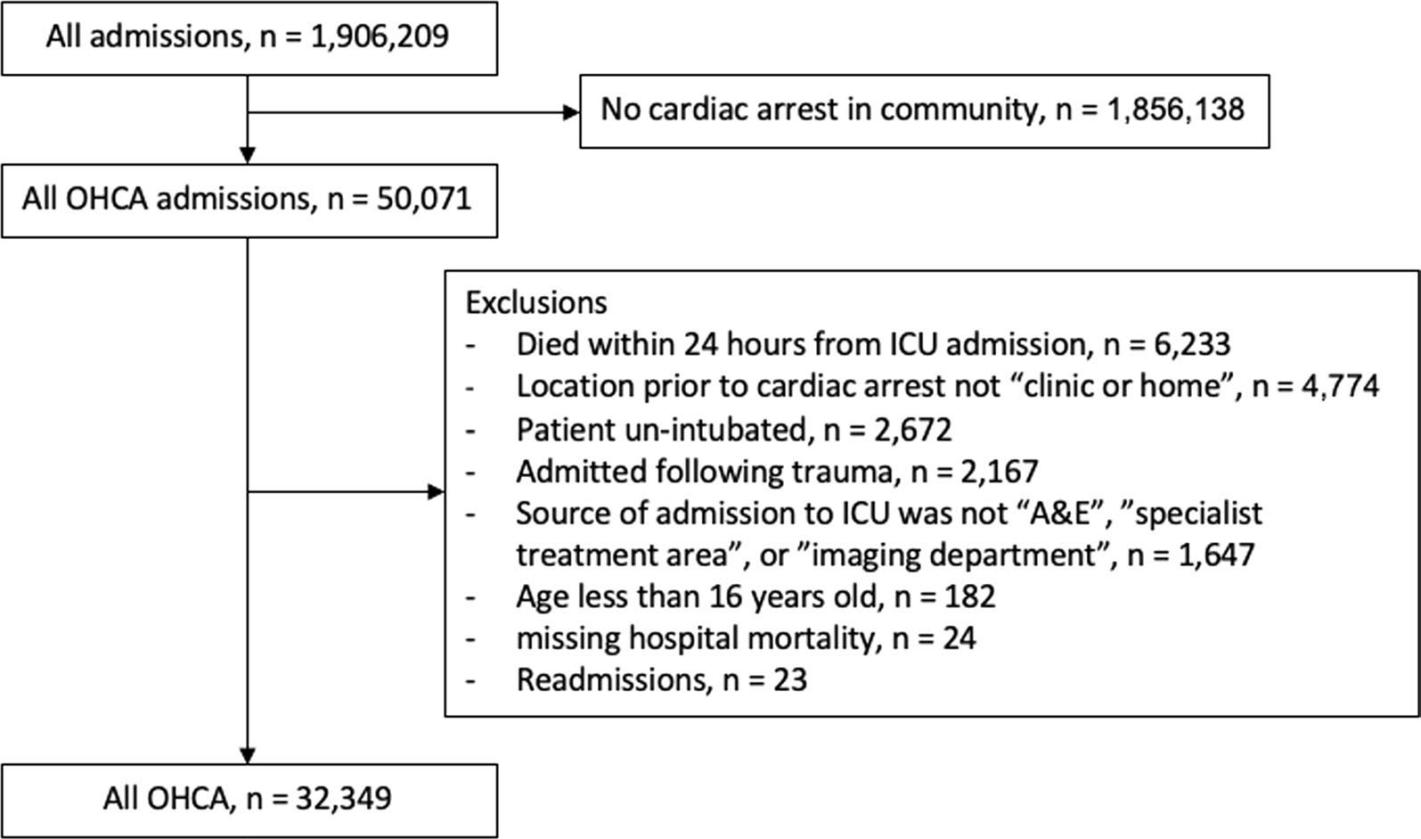
Consort diagram

Over the 10-year study period, a total of 1.9 million patients were included in the ICNARC CMP; after exclusions 32,349 were eligible for inclusion (Fig. 1). No patients had missing blood pressure values. For patients included in the final cohort, 10.3% of variables were missing. Baseline patient demographics are shown in Table 1. The overall hospital mortality was 56.2%. Of those who survived, 87.1% returned to their usual residence.

**Table 1:** Patient demographics

Characteristic	Total, *n* = 32,349	Missing data
Age (years), mean (SD)	60.22 (16.60)	0
Sex, *n* (%)		0
Female	9901 (30.6%)	
Male	22,448 (69.4%)	
Self-reported ethnicity, *n* (%)		1,541 (4.8%)
White	28,132 (91.3%)	
Mixed	205 (0.7%)	
Asian	1371 (4.5%)	
Black	587 (1.9%)	
Other	513 (1.7%)	
Dependency prior to admission to acute hospital, *n* (%)		369 (1.1%)
Able to live without assistance	25,867 (80.9%)	
Minor assistance	4571 (14.3%)	
Major assistance	1294 (4.0%)	
Total assistance	248 (0.8%)	
Severe comorbidity, *n* (%)		367(1.1%)
No	29,356 (91.8%)	
Yes	2626 (8.2%)	
APACHE II Acute Physiology Score (excluding the MAP component), mean (SD)	11.99 (6.11)	284 (0.9%)
Primary diagnosis category, *n* (%)		0
Sepsis	1279 (4.0%)	
Acute coronary syndrome	13,003 (40.2%)	
Cardiac arrhythmia	12,130 (37.5%)	
Other	5937 (18.4%)	
Year, *n* (%)		0
2010	1846 (5.7%)	
2011	2243 (6.9%)	
2012	2568 (7.9%)	
2013	2834 (8.8%)	
2014	3113 (9.6%)	
2015	3405 (10.5%)	
2016	3616 (11.2%)	
2017	3820 (11.8%)	
2018	4326 (13.4%)	
2019	4578 (14.2%)	
Highest serum glucose, mean (SD)	11.72 (5.32)	1210 (3.7%)
Lowest serum glucose, mean (SD)	6.49 (2.51)	1210 (3.7%)
Highest central temperature, mean (SD)	36.74 (1.30)	19 (0.1%)
Lowest central temperature, mean (SD)	34.14 (1.61)	19 (0.1%)
Received advanced cardiovascular support*		0
No	11,995 (37.1%)	
Yes	20,354 (62.9%)	
Treatment withheld/withdrawn, *n* (%)		0
Neither	20,350 (62.9%)	
Withdrawn	9162 (28.3%)	
Withheld	806 (2.5%)	
Both withdrawn and withheld	2031 (6.3%)	
ICU mortality, *n* (%)		0
Alive	17,608 (54.4%)	
Dead	14,741 (45.6%)	
ICU length of stay (days), median (IQR)	3.83 (2.15–6.93)	0
Hospital mortality, *n* (%)		106 (0.3%)
Alive	14,123 (43.8%)	
Dead	18,120 (56.2%)	
Hospital length of stay (days), median (IQR)	7.00 (3.00–17.00)	149 (0.5%)
Residence after discharge, *n* (%)		2974 (9.2%)
Home or residential place	9,584 (32.6%)	
Nursing home	282 (1.0%)	
Health-related institution	1131 (3.9%)	
Non-health-related institution	103 (0.4%)	
Hospice	82 (0.3%)	
No fixed address	73 (0.2%)	
Dead	18,120 (61.7%)	
Composite of survival and return to usual residence, *n* (%)^#^		2997 (9.3%)
Survived and returned to usual residence	9806 (34.4%)	
Survived and did not return to usual residence	1446 (4.9%)	
Dead	18,120 (61.7%)	

*Advanced cardiovascular, indicated by one or more of the following: admissions receiving multiple intravenous and/or rhythm controlling drugs (e.g. inotropes, amiodarone, nitrates, etc.) (of which, at least one must be vasoactive) when used simultaneously to support or control arterial pressure, cardiac output or organ/tissue perfusion. Admissions receiving continuous observation of cardiac output and derived indices (e.g. with a pulmonary artery catheter, lithium dilution, pulse contour analyses, oesophageal Doppler, impedance, and conductance methods.). Admissions with an intra-aortic balloon pump in place and other assist devices. Admissions with a temporary cardiac pacemaker (valid each day whilst connected for therapeutic reasons to a functioning external pacemaker unit)

^#^Excluding those with missing residence status either before admission or after discharge

**Fig. 2:**
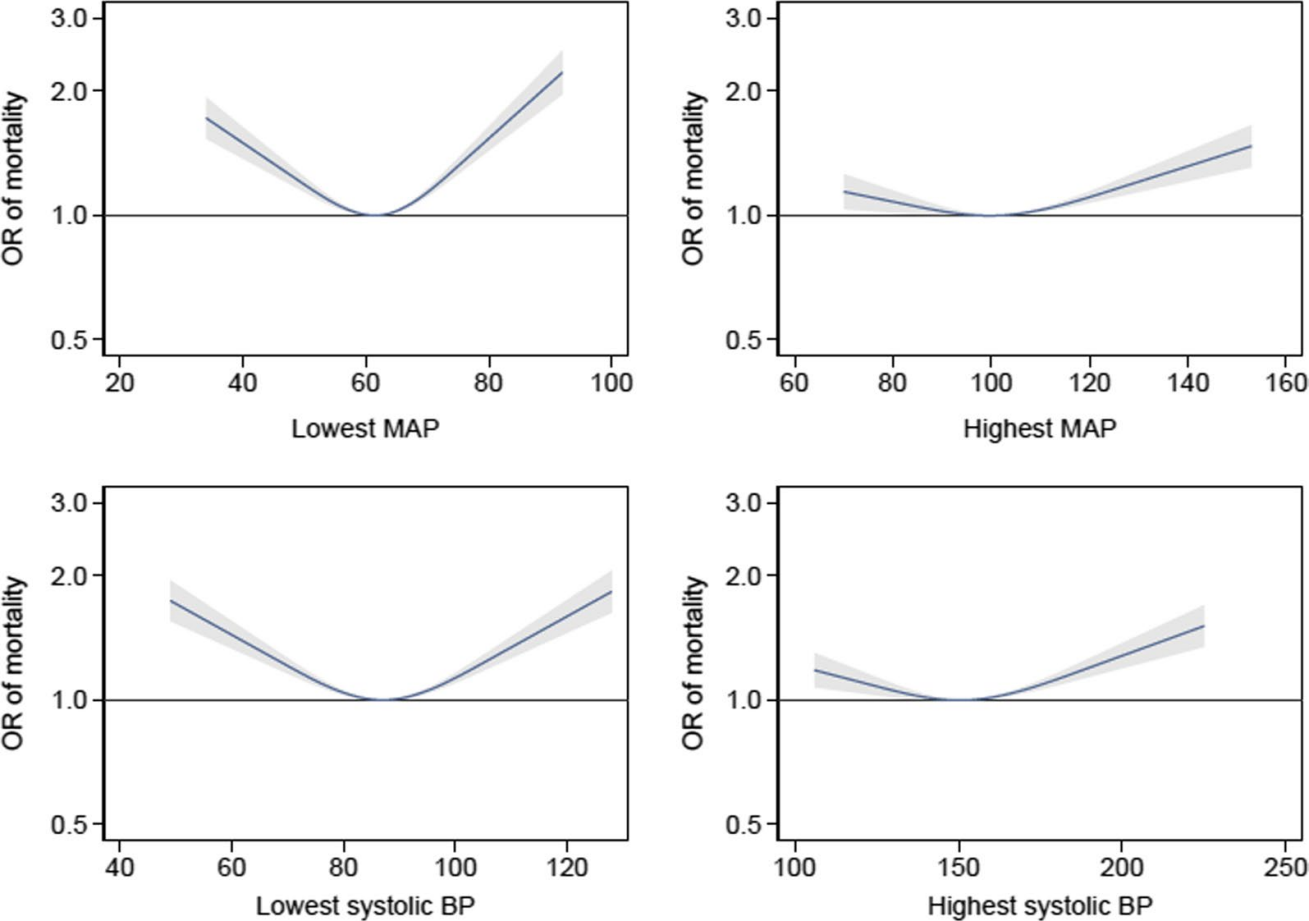
Relationship between blood pressure and mortality. The association between blood pressure and adjusted OR (with 95% CI) for hospital mortality. **Lowest MAP.** The reference point used for calculating mortality odds ratios was patients with a lowest recorded MAP of 62 mmHg. **Highest MAP.** The reference point used for calculating mortality odds ratios was patients with a highest recorded MAP of 102 mmHg. **Lowest SBP.** The reference point used for calculating mortality odds ratios was patients with a lowest recorded SBP of 87 mmHg. **Highest SBP.** The reference point used for calculating mortality odds ratios was patients with a highest recorded SBP of 153 mmHg

Histograms depicting the frequency of lowest and highest blood pressure recordings are presented in Additional file 1: Fig. S1. The relationship between MAP and mortality modelled best using restricted cubic spines with three knots, shown in Fig. 2. Both hypotension and hypertension were associated with mortality.

The median (IQR) lowest recorded MAP for survivors and non-survivors was 63 (57–69) mmHg and 62 (55–69) mmHg, respectively. Lowest MAP demonstrated a U-shaped relationship with mortality. Patients who had a lowest recorded MAP in the range 60–63 mmHg had the lowest associated mortality.

The median (IQR) highest recorded MAP for survivors and non-survivors was 100 (91–112) mmHg and 100 (90–113) mmHg, respectively. Highest MAP demonstrated a J-shaped relationship with mortality. Patients who had a highest recorded MAP in the range 95–104 mmHg had the lowest associated mortality. Adjusted OR for mortality, derived from the restricted cubic splines, for a series of lowest and highest MAP values ranging from the 1st to 99th percentiles is presented in Table 2.

**Table 2:** Relationship between MAP and mortality

MAP (mmHg)	Lowest MAP Adjusted OR (95% CI)	Highest MAP Adjusted OR (95% CI)
35	1.68 (1.51, 1.88)	–
45	1.33 (1.25, 1.42)	–
55	1.07 (1.05, 1.09)	–
62	1 (reference category)	–
65	1.02 (1.02, 1.03)	–
75	1.31 (1.26, 1.37)	1.11 (1.03, 1.20)
85	1.78 (1.63, 1.95)	1.05 (1.00, 1.10)
95	–	1.00 (0.99, 1.02)
102	–	1 (reference category)
105	–	1.01 (1.00, 1.01)
115	–	1.06 (1.04, 1.09)
125	–	1.16 (1.10, 1.21)
135	–	1.26 (1.17, 1.35)
145	–	1.37 (1.25, 1.51)

Adjusted OR with 95% CI for the relationship between lowest and highest MAP and hospital mortality (excluding those who died within the first 24 h). Fields where no values are presented are those beyond the 1st and 99th centiles

The relationship between lowest and highest recorded SBP and mortality followed a similar pattern to MAP, shown in Fig. 2. The median lowest recorded SBP of survivors and non-survivors was 87 (80–95) mmHg and 86 (77–95) mmHg, respectively. Patients who had a lowest recorded SBP in the range 85–89 mmHg had the lowest associated mortality.

The median (IQR) highest recorded SBP of survivors and non-survivors was 150 (135–166) mmHg and 150 (135–169) mmHg, respectively. Patients who had a highest recorded SBP in the range 145–156 mmHg had the lowest associated mortality. Adjusted OR for mortality, derived from the restricted cubic splines, for a series of lowest and highest SBP values ranging from the 1st to 99th percentiles is presented in Table 3.

**Table 3:** Relationship between SBP and mortality

SBP (mmHg)	Lowest SBP Adjusted OR (95% CI)	Highest SBP Adjusted OR (95% CI)
50	1.71 (1.53, 1.91)	–
60	1.44 (1.33, 1.55)	–
70	1.21 (1.16, 1.26)	–
80	1.04 (1.03, 1.06)	–
87	1 (reference category)	–
90	1.01 (1.00, 1.01)	–
100	1.13 (1.10, 1.16)	–
110	1.34 (1.26, 1.42)	1.16 (1.07, 1.27)
120	1.59 (1.45, 1.74)	1.11 (1.04, 1.18)
130	–	1.06 (1.02, 1.10)
140	–	1.02 (1.00, 1.03)
150	–	1.00 (1.00, 1.00)
153	–	1 (reference category)
160	–	1.01 (1.01, 1.02)
170	–	1.06 (1.04–1.08)
180	–	1.12 (1.09–1.16)
190	–	1.20 (1.14–1.26)
200	–	1.28 (1.19–1.37)
210	–	1.37 (1.25–1.50)
220	–	1.46 (1.31–1.63)

Adjusted OR with 95% CI for the relationship between lowest and highest SBP and hospital mortality (excluding those who died within the first 24 h). Fields where no values are presented are those beyond the 1st and 99th centiles

### Sensitivity and post hoc analyses

Sensitivity analyses including those who died within the first 24 h produced results similar to that of the main analysis, Additional file 1: Fig. S2. Missing blood pressure values resulted in the exclusion of 17 patients. Patients who had a lowest recorded MAP in the range 61–64 mmHg had the lowest observed mortality. Patients who had a highest recorded MAP in the range 90–93 mmHg had the lowest observed mortality. Patients who had a lowest recorded SBP in the range 86–90 mmHg had the lowest observed mortality. Patients who had a highest recorded SBP in the range 135–138 mmHg had the lowest observed mortality.

The results of the post hoc analysis with blood pressure treated as a categorical variable are shown in Additional file 1: Tables S2–S9. The blood pressure–mortality relationships typically followed a U-shaped pattern. However, adjusted odds ratios for mortality at the extremes of blood pressure were imprecise. For the lowest recorded MAP, the lowest mortality was observed in the 55–64 mmHg category. For the highest recorded MAP, the lowest mortality was observed in the 105–114 mmHg category. For the lowest recorded SBP, the lowest mortality was observed in the 80–89 mmHg category. Finally, for the highest recorded SBP, the lowest observed mortality encompassed three categories ranging from 130 to 159 mmHg. Two post hoc analyses adjusting for the use of advanced cardiovascular support and using a combined outcome of mortality or failure to return to usual place of residence produced similar results to the primary analysis (Additional file 1: Figs. S3–S4).

## Discussion

In this large retrospective observational study, we found an association between lowest and highest MAP and SBP recorded in the first 24 h of ICU stay and mortality. Hospital mortality was high at 56.2% with nearly one in six survivors unable to return to their usual residence suggesting a high burden of physical or cognitive disability.

Our findings are consistent with evidence showing an association between hypotension and increased mortality [4, 9–20, 27]. In the first 24 h following OHCA, over half of all OHCA patients in the UK experience hypotension below the currently guideline thresholds of MAP 65 mmHg and SBP 90 mmHg [4, 8]. A lowest recorded MAP of 60–63 mmHg and lowest recorded SBP of 85–89 mmHg were associated with the lowest mortality. The blood pressures associated with lowest mortality were below current guideline thresholds of MAP 65 mmHg and SBP 90 mmHg [4, 8]. Previous observational studies have shown worse outcomes in patients exposed to blood pressures below MAP 65 mmHg and SBP 90 mmHg [13, 16–19].

In light of the repeated association between hypotension and mortality following OHCA, three phase II trials have compared higher blood pressure targets with usual care. In the NEUROPROTECT trial, patients were randomised to an early goal directed haemodynamic optimisation strategy (targeting a MAP 85–100 and SVO_2_ 65–75% for 36 h) or usual care. There was no difference between groups in the primary outcome measure of percentage of ischaemic voxels with an apparent diffusion coefficient < 650.10^−6^ mm^2^/s on MRI (a feature of hypoxic brain injury) [28]. The COMACARE trial compared a MAP target of 65–75 mmHg with 80–100 mmHg in patients who had suffered a witnessed, shockable OHCA and were mechanically ventilated in ICU. They found no difference in the primary outcome measure of neurone specific enolase at 48 h [29]. The ENDO-RCA trial compared a MAP of 65 mmHg with 72 mmHg using a blood pressure module which was offset to allow MAP targets to be delivered in a blinded manner. They found no difference in soluble thrombomodulin (a biomarker of endothelial integrity), a series of biomarkers or clinical outcomes [30]. The BOX trial is the only phase III trial comparing blood pressure targets in OHCA patients; it compared a MAP target of 77 mmHg with 63 mmHg delivered in a blinded manner. There was no difference between the groups in the primary outcome measure of death or poor neurological outcome at 90 days [31].

The evidence base demonstrating an association between exposure to hypertension and mortality following OHCA is limited. In 2008, the International Liaison Committee on Resuscitation (ILCOR) suggested targeting a MAP of 65–100 mmHg [21]. In our study, the median highest recorded MAP was 100 mmHg suggesting half of all patients had a MAP above the ILCOR target range. We found that patients who had a highest recorded MAP in the range 95–104 mmHg had the lowest mortality.

Whilst both lowest MAP and SBP demonstrated a U-shaped relationship with mortality, highest recorded MAP and SBP demonstrated a J-shaped relationship with mortality with high mortality seen at the extremes of highest recorded blood pressure. Cerebral oxygen saturations measured by near infrared spectroscopy have been shown to fall with a MAP above 101 mmHg. It is postulated that higher blood pressures may activate cardio-depressive feedback loops due to excessive afterload [9]. Alternatively, extremes of hypertension may represent patients demonstrating a Cushing’s reflex as part of a cerebral herniation syndrome. Our findings are in contrast to Bro-Jeppesen and colleagues who found mortality continued to fall beyond a MAP 110 mmHg when examining the effect of mean MAP averaged over the first 36 h in ICU following OHCA [12, 17]. Haung and colleagues found a MAP of 84–110 mmHg to be associated with favourable neurological outcome [14].

The relationship between blood pressure and mortality following OHCA is complex; we observed an association between exposure to both hypotension and hypertension and increased mortality. Indeed, we observed an increase in mortality in those with a lowest MAP of 85 mmHg, within the target range used in previous phase II trials [28, 29]. We demonstrated the median blood pressure of survivors and non-survivors to be clinically indistinguishable suggesting a heterogeneity of response following exposure to hypotension or hypertension.

Hypoxic ischaemic brain injury after cardiac arrest may result in loss or “rightward shift” of cerebral autoregulation [5, 27]. Loss of cerebral autoregulation may render cerebral blood flow dependent on blood pressure with hypotension resulting in further cerebral ischaemia and hypertension risking cerebral oedema [6, 32]. Following OHCA, individual patients demonstrate a wide range in cerebral autoregulatory thresholds [33], with preserved autoregulation an independent predictor of survival [34]. Targeting a higher blood pressure without knowledge of an individual patient’s autoregulatory thresholds may expose them to either hypoperfusion or hyperperfusion. This may explain why the BOX trial [31] and previous phase II trials [28–30] did not demonstrate a difference in mortality or neurological outcomes when comparing different blood pressure targets. An individualised blood pressure management strategy, guided by neuromonitoring to demonstrate limits of cerebral autoregulation, may be more appropriate than a “one-size-fits-all” blood pressure strategy [34]. The European Resuscitation Council has called for “research into identification of optimal MAP targets for individual cardiac arrest survivors receiving intensive care” [4]. Individual blood pressure targets have successfully been derived in observational studies in OHCA [35, 36]. The COGiTATE trial has demonstrated that targeting optimal blood pressure is safe and feasible in patients with traumatic brain injury [37]. Our planned phase II randomised controlled trial will look at individualised blood pressure targets following OHCA.

Our study had a number of strengths and limitations. The ICNARC CMP data are collected from across a large number of ICUs in the UK and therefore represents a generalisable population of patients following OHCA. Data undergo validation prior to entry in the database. We included a homogenous population of OHCA patients unlike other studies which included a mixed cohort of IHCA and OHCA [9, 11, 32]. We examined the effect of exposure to hypotension, as evidenced by lowest recorded MAP and SBP, reflecting international guidelines [4, 8]. Other studies have examined blood pressure in the first 6 hours [10, 13, 38], at fixed time points in the first 24 h [11, 14, 27] or during periods of haemodynamic stability [20]. Therefore, previous studies may have risked missing episodes of hypotension. The findings of the MAP and SBP analysis are concordant and are supported by sensitivity and post hoc analyses.

As a retrospective study, we cannot infer causality. The association between blood pressure and mortality may reflect residual confounding factors including prolonged duration of cardiac arrest, more severe post cardiac arrest syndrome, or loss of cerebral autoregulation. Unfortunately, the ICNARC CMP data do not record intra-arrest characteristics. Therefore, we were unable to adjust for important prognostic features including presenting rhythm, bystander intervention, or duration of OHCA [39]. However, we adjusted for illness severity using a modified acute physiology component of the APACHE II score, an approach that is well established [3, 40, 41] and validated in an OHCA population [42]. The ability of APACHE II score to predict mortality following OHCA is similar to that of the disease specific OHCA score [43].

In clinical practice, prolonged periods of hypotension may be more deleterious than brief periods of hypotension. Exposure to hypotension measured as an integral of blood pressure and time below a threshold has previously been associated with both mortality and poor neurological outcome [13, 18]. We were unable to quantify the duration of hypotension as this is not recorded the ICNARC CMP data and thus were unable to test this hypothesis. The ICNARC CMP does not record serial blood pressure values; therefore, we are unable to describe the temporal changes in blood pressure in our cohort. Nor, can we determine whether exposure to hypotension in the first 6 h in ICU, as investigated by others, is of more prognostic significance than blood pressure as recorded in our study [38]. We were unable to differentiate between those with intact or lost cerebral autoregulation. We were also unable to examine the interaction between hypotension and vasoactive exposure as the ICNARC CMP did not collect detailed information on vasopressor use. To address this, we presented a post hoc analysis adjusting for advanced cardiovascular support. However, even after adjustment for vasopressor exposure an association between MAP and mortality has been demonstrated [12]. Previous studies have demonstrated that patients cooled to 33 °C have greater vasopressor requirements than those cooled to 36 °C. [12] Whilst we corrected for highest recorded temperature, we were unable to adjust for target temperature. We could not adjust for pre-existing hypertension as this is not recorded in the ICNARC CMP. Finally, we were unable to report on neurological outcome and therefore presented analysis using a combined outcome of mortality or failure to return to usual place of residence as a surrogate for poor neurological outcome.

In conclusion, we found an association between exposure to hypotension and hypertension in the first 24 h of ICU care and increased mortality following OHCA. The inability to distinguish between the median blood pressure of survivors and non-survivors reinforces the need for research into individualised blood pressure targets for survivors following OHCA.

### Supplementary Information

**Additional file 1:** Supplementary Appendix.

## Data Availability

The data that support the findings of this study are available from ICNARC but restrictions apply to the availability of these data, which were used under license for the current study, and so are not publicly available. Data are, however, available from the authors upon reasonable request and with permission of ICNARC’s independent Data Access Advisory Group.

## Abbreviations

APACHE: Acute Physiology and Chronic Health Evaluation
CMP: Case mix programme
CPP: Cerebral perfusion pressure
CPR: Cardiopulmonary resuscitation
DBP: Diastolic blood pressure
ICNARC: Intensive Care National Audit and Research Centre
ICU: Intensive care unit
IHCA: In hospital cardiac arrest
IQR: Interquartile range
MAP: Mean arterial pressure
OHCA: Out-of-hospital cardiac arrest
SBP: Systolic blood pressure

## References

[1] Nolan JP, Orzechowska I, Harrison DA, Soar J, Perkins GD, Shankar-Hari M (2021). Changes in temperature management and outcome after out-of-hospital cardiac arrest in United Kingdom intensive care units following publication of the targeted temperature management trial. Resuscitation.

[2] Hawkes C, Booth S, Ji C, Brace-McDonnell SJ, Whittington A, Mapstone J (2017). Epidemiology and outcomes from out-of-hospital cardiac arrests in England. Resuscitation.

[3] McGuigan PJ, Shankar-Hari M, Harrison DA, Laffey JG, McAuley DF (2020). The interaction between arterial oxygenation and carbon dioxide and hospital mortality following out of hospital cardiac arrest: a cohort study. Crit Care.

[4] Nolan JP, Sandroni C, Böttiger BW (2021). European Resuscitation Council and European Society of Intensive Care Medicine guidelines 2021: post-resuscitation care. Intensive Care Med.

[5] Sundgreen C, Larsen FS, Herzog TM, Knudsen GM, Boesgaard S, Aldershvile J (2001). Autoregulation of cerebral blood flow in patients resuscitated from cardiac arrest. Stroke.

[6] Sekhon MS, Ainslie PN, Griesdale DE (2017). Clinical pathophysiology of hypoxic ischemic brain injury after cardiac arrest: a “two-hit” model. Crit Care.

[7] Stub D, Bernard S, Duffy SJ, Kaye DM (2011). Post cardiac arrest syndrome. Circulation.

[8] Panchal AR, Bartos JA, Cabañas JG (2020). Part 3: adult basic and advanced life support: 2020 American Heart Association guidelines for cardiopulmonary resuscitation and emergency cardiovascular care. Circulation.

[9] Ameloot K, Meex I, Genbrugge C, Jans F, Boer W, Verhaert D (2015). Hemodynamic targets during therapeutic hypothermia after cardiac arrest: a prospective observational study. Resuscitation.

[10] Annoni F, Dell’Anna AM, Franchi F, Creteur J, Scolletta S, Vincent JL (2018). The impact of diastolic blood pressure values on the neurological outcome of cardiac arrest patients. Resuscitation.

[11] Beylin ME, Perman SM, Abella BS, Leary M, Shofer FS, Grossestreuer AV (2013). Higher mean arterial pressure with or without vasoactive agents is associated with increased survival and better neurological outcomes in comatose survivors of cardiac arrest. Intensive Care Med.

[12] Bro-Jeppesen J, Annborn M, Hassager C, Wise MP, Pelosi P, Nielsen N (2015). Hemodynamics and vasopressor support during targeted temperature management at 33°c versus 36°c after out-of-hospital cardiac arrest: a post hoc study of the target temperature management trial*. Crit Care Med.

[13] Chiu YK, Lui CT, Tsui KL (2018). Impact of hypotension after return of spontaneous circulation on survival in patients of out-of-hospital cardiac arrest. Am J Emerg Med.

[14] Huang CH, Tsai MS, Ong HN, Chen W, Wang CH, Chang WT (2017). Association of hemodynamic variables with in-hospital mortality and favorable neurological outcomes in post-cardiac arrest care with targeted temperature management. Resuscitation.

[15] Janiczek JA, Winger DG, Coppler P, Sabedra AR, Murray H, Pinsky MR (2016). Hemodynamic resuscitation characteristics associated with improved survival and shock resolution after cardiac arrest. Shock.

[16] Kaji AH, Hanif AM, Thomas JL, Niemann JT (2011). Out-of-hospital cardiac arrest: early in-hospital hypotension versus out-of-hospital factors in predicting in-hospital mortality among those surviving to hospital admission. Resuscitation.

[17] Laurikkala J, Wilkman E, Pettilä V, Kurola J, Reinikainen M, Hoppu S (2016). Mean arterial pressure and vasopressor load after out-of-hospital cardiac arrest: associations with one-year neurologic outcome. Resuscitation.

[18] Russo JJ, Di Santo P, Simard T, James TE, Hibbert B, Couture E (2018). Optimal mean arterial pressure in comatose survivors of out-of-hospital cardiac arrest: an analysis of area below blood pressure thresholds. Resuscitation.

[19] Trzeciak S, Jones AE, Kilgannon JH, Milcarek B, Hunter K, Shapiro NI (2009). Significance of arterial hypotension after resuscitation from cardiac arrest*. Crit Care Med.

[20] Young MN, Hollenbeck RD, Pollock JS, Giuseffi JL, Wang L, Harrell FE (2015). Higher achieved mean arterial pressure during therapeutic hypothermia is not associated with neurologically intact survival following cardiac arrest. Resuscitation.

[21] Neumar RW, Nolan JP, Adrie C, Aibiki M, Berg RA, Böttiger BW (2008). Post-cardiac arrest syndrome: epidemiology, pathophysiology, treatment, and prognostication a consensus statement from the International Liaison Committee on Resuscitation (American Heart Association, Australian and New Zealand Council on Resuscitation, European Resuscitation Council, Heart and Stroke Foundation of Canada, InterAmerican Heart Foundation, Resuscitation Council of Asia, and the Resuscitation Council of Southern Africa); the American Heart Association Emergency Cardiovascular Care Committee; the Council on Cardiovascular Surgery and Anesthesia; the Council on Cardiopulmonary, Perioperative, and Critical Care; the Council on Clinical Cardiology; and the Stroke Council. Circulation.

[22] Ferrando-Vivas P, Jones A, Rowan KM, Harrison DA (2017). Development and validation of the new ICNARC model for prediction of acute hospital mortality in adult critical care. J Crit Care.

[23] Elmer J, Scutella M, Pullalarevu R (2015). The association between hyperoxia and patient outcomes after cardiac arrest: analysis of a high-resolution database. Intensive Care Med.

[24] Lee BK, Jeung KW, Lee HY (2014). Association between mean arterial blood gas tension and outcome in cardiac arrest patients treated with therapeutic hypothermia. Am J Emerg Med.

[25] Elmer J, Torres C, Aufderheide TP, Austin MA, Callaway CW, Golan E (2016). Association of early withdrawal of life-sustaining therapy for perceived neurological prognosis with mortality after cardiac arrest. Resuscitation.

[26] Althouse AD (2016). Adjust for multiple comparisons? It’s not that simple. Ann Thorac Surg.

[27] Bhate TD, McDonald B, Sekhon MS, Griesdale DEG (2015). Association between blood pressure and outcomes in patients after cardiac arrest: a systematic review. Resuscitation.

[28] Ameloot K, De Deyne C, Eertmans W (2019). Early goal-directed haemodynamic optimization of cerebral oxygenation in comatose survivors after cardiac arrest: the Neuroprotect post-cardiac arrest trial. Eur Heart J.

[29] Jakkula P, Pettilä V, Skrifvars MB (2018). Targeting low-normal or high-normal mean arterial pressure after cardiac arrest and resuscitation: a randomised pilot trial. Intensive Care Med.

[30] Grand J, Meyer AS, Kjaergaard J (2020). A randomised double-blind pilot trial comparing a mean arterial pressure target of 65 mm Hg versus 72 mm Hg after out-of-hospital cardiac arrest. Eur Heart J Acute Cardiovasc Care.

[31] Kjaergaard J, Møller JE, Schmidt H, Grand J, Mølstrøm S, Borregaard B (2022). Blood-pressure targets in comatose survivors of cardiac arrest. N Engl J Med.

[32] Roberts BW, Kilgannon JH, Hunter BR, Puskarich MA, Shea L, Donnino M (2019). Association between elevated mean arterial blood pressure and neurologic outcome after resuscitation from cardiac arrest: results from a multicenter prospective cohort study*. Crit Care Med.

[33] Rikhraj KJK, Wood MD, Hoiland RL, Thiara S, Griesdale DEG, Sekhon MS (2021). Determining optimal mean arterial pressure after cardiac arrest: a systematic review. Neurocrit Care.

[34] Ameloot K, Genbrugge C, Meex I, Jans F, Boer W, Vander Laenen M (2015). An observational near-infrared spectroscopy study on cerebral autoregulation in post-cardiac arrest patients: time to drop “one-size-fits-all” hemodynamic targets?. Resuscitation.

[35] Pham P, Bindra J, Chuan A, Jaeger M, Aneman A (2015). Are changes in cerebrovascular autoregulation following cardiac arrest associated with neurological outcome? Results of a pilot study. Resuscitation.

[36] Sekhon MS, Smielewski P, Bhate TD (2016). Using the relationship between brain tissue regional saturation of oxygen and mean arterial pressure to determine the optimal mean arterial pressure in patients following cardiac arrest: a pilot proof-of-concept study. Resuscitation.

[37] Tas J, Beqiri E, van Kaam RC, Czosnyka M, Donnelly J, Haeren RH (2021). Targeting autoregulation-guided cerebral perfusion pressure after traumatic brain injury (COGiTATE): a feasibility randomized controlled clinical trial. J Neurotrauma.

[38] Kilgannon JH, Roberts BW, Jones AE, Mittal N, Cohen E, Mitchell J (2014). Arterial blood pressure and neurologic outcome after resuscitation from cardiac arrest*. Crit Care Med.

[39] Hasselqvist-Ax I, Riva G, Herlitz J (2015). Early cardiopulmonary resuscitation in out-of-hospital cardiac arrest. N Engl J Med.

[40] Bellomo R, Bailey M, Eastwood GM (2011). Arterial hyperoxia and in-hospital mortality after resuscitation from cardiac arrest. Crit Care.

[41] Schneider AG, Eastwood GM, Bellomo R (2013). Arterial carbon dioxide tension and outcome in patients admitted to the intensive care unit after cardiac arrest. Resuscitation.

[42] Niskanen M, Kari A, Nikki P, Iisalo E, Kaukinen L, Rauhala V (1991). Acute Physiology and Chronic Health Evaluation (APACHE II) and Glasgow Coma Scores as predictors of outcome from intensive care after cardiac arrest. Crit Care Med.

[43] Isenschmid C, Luescher T, Rasiah R, Kalt J, Tondorf T, Gamp M (2019). Performance of clinical risk scores to predict mortality and neurological outcome in cardiac arrest patients. Resuscitation.

