# Publisher Correction: The effect of blood pressure on mortality following out‑of‑hospital cardiac arrest: a retrospective cohort study of the United Kingdom Intensive Care National Audit and Research Centre database

**DOI:** 10.1186/s13054-023-04458-x

**Published:** 2023-05-04

**Authors:** Peter J. McGuigan, Elisa Giallongo, Bronagh Blackwood, James Doidge, David A. Harrison, Alistair D. Nichol, Kathryn M. Rowan, Manu Shankar‑Hari, Markus B. Skrifvars, Karen Thomas, Danny F. McAuley

**Affiliations:** 1 Regional Intensive Care Unit, Royal Victoria Hospital, Belfast, UK; 2 Wellcome‑Wolfson Institute for Experimental Medicine, Queen’s University, Belfast, UK; 3 Intensive Care National Audit and Research Centre, Napier House, 24 High Holborn, London, UK; 4 University College Dublin Clinical Research Centre, St Vincent’s University Hospital, Dublin, Ireland; 5 The Australian and New Zealand Intensive Care Research Centre, Monash University, Melbourne, Australia; 6 The Alfred Hospital, Melbourne, Australia; 7 Centre for Inflammation Research, Institute of Regeneration and Repair, University of Edinburgh, Edinburgh, UK; 8 Royal Infirmary of Edinburgh, NHS Lothian, Edinburgh, UK; 9 Department of Emergency Care and Services, University of Helsinki, Helsinki, Finland; 10 Helsinki University Hospital, Helsinki, Finland

**Correction : Crit Care (2023) 27:4** **https://doi.org/10.1186/s13054-022-04289-2**

Following publication of the original article [1], the authors identified that the corresponding author was indicated twice in the author group.

The publisher apologizes for the inconvenience caused.

The author group has been updated above and the original article [1] has been corrected.
