# Supplementary material for: The effect of blood pressure on mortality following out-of-hospital cardiac arrest: a retrospective cohort study of the United Kingdom Intensive Care National Audit and Research Centre database
Source: Crit Care. 2023 Jan 5;27:4. doi: 10.1186/s13054-022-04289-2 (PMC9817239; doi:10.1186/s13054-022-04289-2)
Supplement: Supplementary file 1 — Additional file 1: Supplementary Appendix. [file 13054_2022_4289_MOESM1_ESM.docx]

**Additional File 1.**

**Table 1. Variables included in logistic regression analysis**

|  |  | **Lowest MAP spline model (n=32,349)** | | **Highest MAP spline model (n=32,349)** | | **Lowest SBP spline model (n=32,349)** | | **Highest SBP spline model (n=32,349)** | |
| --- | --- | --- | --- | --- | --- | --- | --- | --- | --- |
|  | **Unadjusted OR**  **(95% CI)** | **Adjusted OR**  **(95% CI)** | **p-value** | **Adjusted OR**  **(95% CI)** | **p-value** | **Adjusted OR**  **(95% CI)** | **p-value** | **Adjusted OR**  **(95% CI)** | **p-value** |
| **Age*** | 1.02 (1.02-1.02) | 1.02 (1.021.02) | <0.001 | 1.02 (1.02-1.02) | <0.001 | 1.02 (1.02-1.02) | <0.001 | 1.02 (1.02-1.02) | <0.001 |
| **Sex** |  |  |  |  |  |  |  |  |  |
| Female | 1 (reference category) | 1 (reference category) |  | 1 (reference category) |  | 1 (reference category) |  | 1 (reference category) |  |
| Male | 0.69 (0.66-0.73) | 0.77 (0.73-0.82) | <0.001 | 0.77 (0.73-0.81) | <0.001 | 0.78 (0.74-0.82) | <0.001 | 0.77 (0.73-0.81) | <0.001 |
| **Self-reported ethnicity** |  |  |  |  |  |  |  |  |  |
| White | 1 (reference category) | 1 (reference category) |  | 1 (reference category) |  | 1 (reference category) |  | 1 (reference category) |  |
| Mixed | 0.84 (0.64-1.11) | 0.95 (0.70-1.31) | 0.768 | 0.93 (0.68-1.28) | 0.665 | 0.94 (0.69-1.29) | 0.720 | 0.93 (0.68-1.28) | 0.667 |
| Asian | 1.21 (1.08-1.35) | 1.13 (1.00-1.27) | 0.052 | 1.14 (1.01-1.28) | 0.040 | 1.12 (0.99-1.26) | 0.069 | 1.13 (1.01-1.28) | 0.040 |
| Black | 1.00 (0.84-1.17) | 0.85 (0.70-1.02) | 0.083 | 0.86 (0.72-1.04) | 0.121 | 0.86 (0.71-1.03) | 0.110 | 0.86 (0.71-1.03) | 0.110 |
| Other | 1.02 (0.86-1.22) | 1.15 (0.95-1.40) | 0.159 | 1.15 (0.94-1.39) | 0.167 | 1.14 (0.94-1.38) | 0.188 | 1.14 (0.94-1.39) | 0.178 |
| **Dependency prior to hospital admission** |  |  |  |  |  |  |  |  |  |
| Able to live without assistance | 1 (reference category) | 1 (reference category) |  | 1 (reference category) |  | 1 (reference category) |  |  |  |
| Minor assistance | 2.19 (2.04-2.34) | 1.66 (1.54-1.79) | <0.001 | 1.66 (1.53-1.79) | <0.001 | 1.66 (1.53-.179) | <0.001 | 1.65 (1.53-1.78) | <0.001 |
| Major assistance | 3.66 (3.19-4.21) | 2.77 (2.39-3.22) | <0.001 | 2.76 (2.38-3.20) | <0.001 | 2.77 (2.38-3.21) | <0.001 | 2.75 (2.37-3.19) | <0.001 |
| Total assistance | 2.86 (2.14-3.83) | 3.07 (2.24-4.19) | <0.001 | 3.00 (2.20-4.09) | <0.001 | 3.02 (2.21-4.13) | <0.001 | 2.99 (2.19-4.08) | <0.001 |
| **APACHE II severe co-morbidity** |  |  |  |  |  |  |  |  |  |
| No | 1 (reference category) | 1 (reference category) |  | 1 (reference category) |  | 1 (reference category) |  | 1 (reference category) |  |
| Yes | 1.92 (1.76-2.10) | 1.02 (1.00-1.04) | 0.080 | 1.02 (1.00-1.04) | 0.077 | 1.02 (1.00-1.04) | 0.099 | 1.02 (1.00-1.04) | 0.088 |
| **APACHE II Acute Physiology Score (excluding MAP)^#^** | 1.12 (1.11-1.12) | 1.11 (1.11-1.12) | <0.001 | 1.11 (1.11-1.12) | <0.001 | 1.11(1.11-1.12) | <0.001 | 1.11 (1.11-1.12) | <0.001 |
| **Primary diagnosis category** |  |  |  |  |  |  |  |  |  |
| Sepsis | 1 (reference category) | 1 (reference category) |  | 1 (reference category) |  | 1 (reference category) |  | 1 (reference category) |  |
| Acute coronary syndrome | 0.91 (0.81-1.02) | 1.22 (1.07-1.38) | 0.003 | 1.20 (1.05-1.36) | 0.006 | 1.22 (1.07-1.38) | 0.003 | 1.20 (1.05-1.36) | 0.006 |
| Cardiac arrhythmia | 0.83 (0.74-0.93) | 1.07 (0.94-1.22) | 0.279 | 1.06 (0.93-1.20) | 0.386 | 1.07 (0.94-1.22) | 0.285 | 1.06 (0.93-1.20) | 0.381 |
| Other | 1.35 (1.19-1.52) | 2.07 (1.81-2.37) | <0.001 | 2.08 (1.82-2.39) | <0.001 | 2.09 (1.82-2.39) | <0.001 | 2.08 (1.82-2.39) | <0.001 |
| **Year^** | 1.01 (1.00-1.02) | 1.04 (1.03-1.05) | <0.001 | 1.04 (1.03-1.05) | <0.001 | 1.04 (1.03-1.05) | <0.001 | 1.04 (1.03-1.05) | <0.001 |
| **Highest central temperature^¶^** | 0.96 (0.94-0.98) | 0.93 (0.92-0.95) | <0.001 | 0.94 (0.92-0.95) | <0.001 | 0.93 (0.92-0.95) | <0.001 | 0.93 (0.92-0.95) | <0.001 |
| **Lowest serum glucose†** | 1.10 (1.08-1.11) | 1.06 (1.05-1.08) | <0.001 | 1.07 (1.06-1.08) | <0.001 | 1.07 (1.05-1.08) | <0.001 | 1.07 (1.06-1.08) | <0.001 |

* per one-year increase in age

^#^ per point increase in APACHE II acute physiology score

^ per year

¶ per ^o^C

† per mmol/L

**Figure 1.** Distribution of lowest and highest recorded MAP and SBP amongst survivors and non-survivors (excluding those who died within the first 24 hours)

**Sensitivity analysis (including those who died within the first 24 hours)**

**Figure 2.** Adjusted OR with 95% CI for the relationship between blood pressure and hospital mortality (including those who died within the first 24 hours).


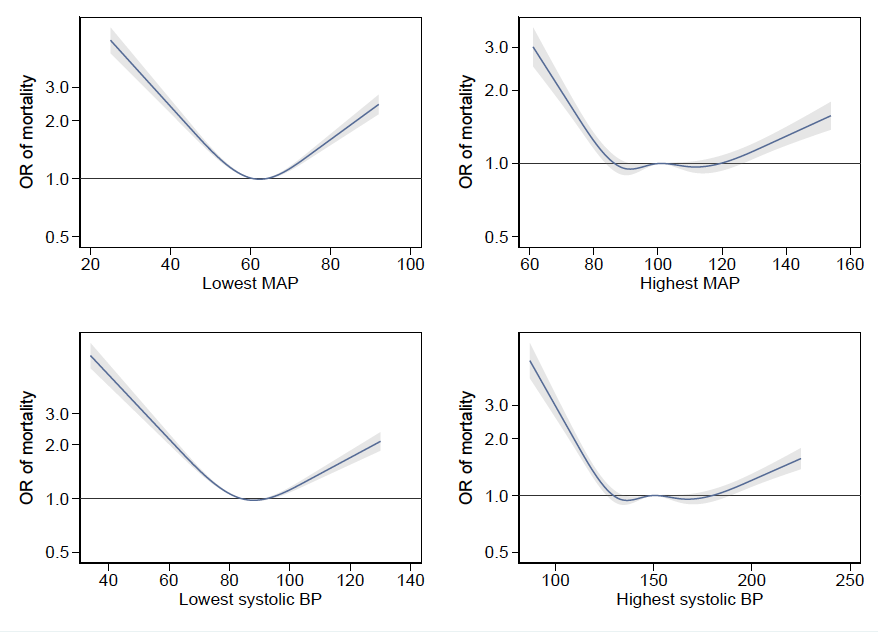


Lowest MAP was modelled by unrestricted cubic splines with three knots at the 10^th^, 50^th^ and 90^th^ percentiles (equating to 45mmHg, 61.7mmHg, and 74.7mmHg respectively) using a logistic regression model. The reference point used for calculating mortality odds ratios was patients with a lowest recorded MAP of 61mmHg. The lowest mortality was observed between 61-64mmHg.

Highest MAP was modelled by unrestricted cubic splines with five knots at the 5^th^, 27.5^th^, 50^th^, 72.5^th^ and 95^th^ percentiles (equating to 74mmHg, 89.7mmHg, 99mmHg, 110mmHg, and 133.7mmHg respectively) using a logistic regression model. The reference point used for calculating mortality odds ratios was patients with a highest recorded MAP of 101mmHg. The lowest mortality was observed between 90-93mmHg.

Lowest SBP was modelled by unrestricted cubic splines with three knots at the 10^th^, 50^th^ and 90^th^ percentiles (equating to 62mmHg, 85mmHg, and 105mmHg respectively) using a logistic regression model. The reference point used for calculating mortality odds ratios was patients with a lowest recorded SBP of 84mmHg. The lowest mortality was observed between 86-90mmHg.

Highest SBP was modelled by unrestricted cubic splines with five knots at the 5^th^, 27.5^th^, 50^th^, 72.5^th^ and 95^th^ percentiles (equating to 110mmHg, 133mmHg, 148mmHg, 164mmHg, and 200mmHg respectively) using a logistic regression model. The reference point used for calculating mortality odds ratios was patients with a highest recorded SBP of 150mmHg. The lowest mortality was observed between 135-138mmHg.

**Post hoc analysis (excluding those who died within the first 24 hours)**

**Figure 3.** Adjusted OR with 95% CI for the relationship between blood pressure and hospital mortality (excluding those who died within the first 24 hours), adjusted for the use of advanced cardiovascular support*

***** Advanced Cardiovascular, indicated by one or more of the following:

- admissions receiving multiple intravenous and/or rhythm controlling drugs (e.g. inotropes, amiodarone, nitrates etc.) (of which, at least one must be vasoactive) when used simultaneously to support or control arterial pressure, cardiac output or organ/tissue perfusion
- admissions receiving continuous observation of cardiac output and derived indices (e.g. with a pulmonary artery catheter, lithium dilution, pulse contour analyses, oesophageal doppler, impedance and conductance methods.)
- admissions with an intra-aortic balloon pump in place and other assist devices
- admissions with a temporary cardiac pacemaker (valid each day while connected for therapeutic reasons to a functioning external pacemaker unit)


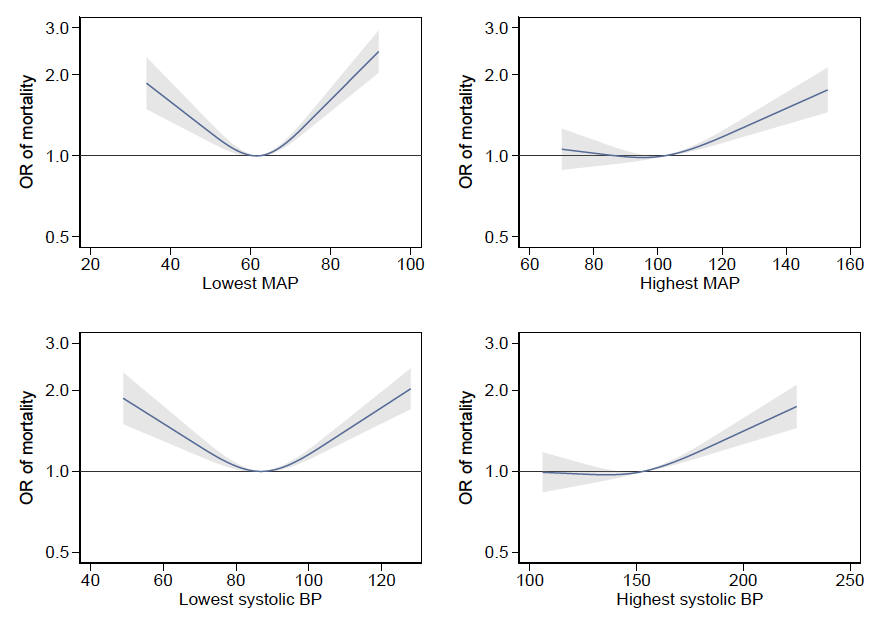


Lowest MAP. The reference point used for calculating mortality odds ratios was patients with a lowest recorded MAP of 62mmHg. The lowest mortality was observed between 60-63mmHg.

Highest MAP. The reference point used for calculating mortality odds ratios was patients with a highest recorded MAP of 102mmHg. The lowest mortality was observed between 89-100mmHg.

Lowest SBP. The reference point used for calculating mortality odds ratios was patients with a lowest recorded SBP of 87mmHg. The lowest mortality was observed between 85-89mmHg.

Highest SBP. The reference point used for calculating mortality odds ratios was patients with a highest recorded SBP of 153mmHg. The lowest mortality was observed between 128-142mmHg.

**Figure 4.** Adjusted OR with 95% CI for the relationship between blood pressure and a composite outcome of mortality or failure to return to usual place of residence (excluding those who died within the first 24 hours), adjusted for the use of advanced cardiovascular support.


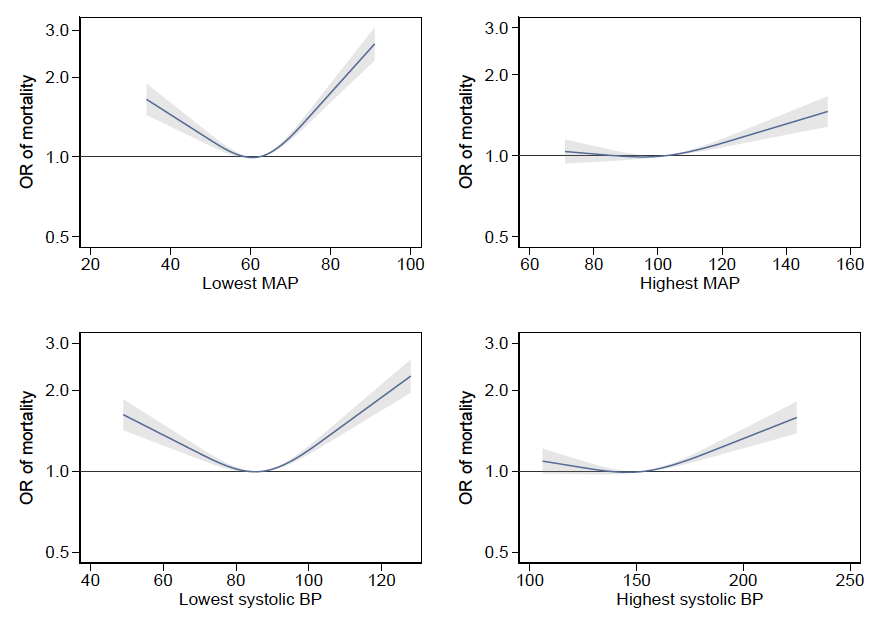


Lowest MAP. The reference point used for calculating odds ratios for mortality or failure to return to usual place of residence was patients with a lowest recorded MAP of 62mmHg. Rates of mortality or failure to return to usual place of residence were lowest at 58-62mmHg.

Highest MAP. The reference point used for calculating odds ratios for mortality or failure to return to usual place of residence was patients with a highest recorded MAP of 102mmHg. Rates of mortality or failure to return to usual place of residence were lowest at 90-99mmHg.

Lowest SBP. The reference point used for calculating odds ratios for mortality or failure to return to usual place of residence was patients with a lowest recorded SBP of 87mmHg. Rates of mortality or failure to return to usual place of residence were lowest at 83-88mmHg.

Highest SBP. The reference point used for calculating odds ratios for mortality or failure to return to usual place of residence was patients with a highest recorded SBP of 153mmHg. Rates of mortality or failure to return to usual place of residence were lowest at 142-150mmHg.

**Post hoc analysis with blood pressure as a categorical variable (excluding those who died within the first 24 hours)**

**Table 2.** Unadjusted and adjusted OR with 95% CI for the relationship between lowest MAP and hospital mortality (excluding those who died within the first 24 hours) when blood pressure was modelled as a categorical variable.

*Categories where no OR for mortality is presented are those where there were either zero survivors, zero non-survivors or zero patients.*

| **Lowest MAP** | **Survivors**  **n=14,123** | **Non-survivors**  **n=18,120** | **Unadjusted OR for mortality (95% CI)** | **Adjusted OR for mortality (95% CI)** | **p-value** |
| --- | --- | --- | --- | --- | --- |
| <45 | 499 (3.5%) | 1,238 (6.8%) | 2.22 (1.99, 2.48) | 1.53 (1.36, 1.73) | <0.001 |
| 45-54 | 2,059 (14.6%) | 3,005 (16.6%) | 1.31 (1.22, 1.40) | 1.02 (0.95, 1.10) | 0.539 |
| 55-64 | 5,695 (40.3%) | 6,699 (37.0%) | 1.05 (1.00, 1.11) | 0.93 (0.88, 0.99) | 0.015 |
| 65-74 (baseline) | 4,582 (32.4%) | 5,116 (28.2%) | 1.00 | 1.00 | - |
| 75-84 | 1,001 (7.1%) | 1,569 (8.7%) | 1.40 (1.28, 1.53) | 1.48 (1.34, 1.62) | <0.001 |
| 85-94 | 223 (1.6%) | 369 (2.0%) | 1.48 (1.25, 1.76) | 1.68 (1.40, 2.02) | <0.001 |
| 95-104 | 46 (0.3%) | 84 (0.5%) | 1.64 (1.14, 2.35) | 1.77 (1.20, 2.62) | 0.004 |
| 105-114 | 15 (0.1%) | 30 (0.2%) | 1.79 (0.96, 3.33) | 1.74 (0.89, 3.41) | 0.107 |
| 115-124 | 2 (0.0%) | 9 (0.0%) | 4.03 (0.87, 18.66) | 2.96 (0.56, 15.65) | 0.202 |
| 125-134 | 1 (0.0%) | 0 (0.0%) | - | - | - |
| 134-145 | 0 (0.0%) | 0 (0.0%) | - | - | - |
| 145>= | 0 (0.0%) | 1 (0.0%) | - | - | - |

**Table 3.** Unadjusted and adjusted OR with 95% CI for the relationship between highest MAP and hospital mortality (excluding those who died within the first 24 hours) when blood pressure was modelled as a categorical variable.

*Categories where no OR for mortality is presented are those where there were either zero survivors, zero non-survivors or zero patients.*

| **Highest MAP** | **Survivors**  **n=14,123** | **Non-survivors**  **n=18,120** | **Unadjusted OR for mortality (95% CI)** | **Adjusted OR for mortality (95% CI)** | **p-value** |
| --- | --- | --- | --- | --- | --- |
| <45 | 0 (0.0%) | 1 (0.0%) | - | - | - |
| 45-54 | 1 (0.0%) | 7 (0.0%) | 5.25 (0.65, 42.67) | 4.49 (0.52, 38.46) | 0.171 |
| 55-64 | 22 (0.2%) | 46 (0.3%) | 1.57 (0.94, 2.61) | 1.26 (0.72, 2.18) | 0.421 |
| 65-74 | 277 (2.0%) | 450 (2.5%) | 1.22 (1.04, 1.42) | 1.08 (0.91, 1.28) | 0.382 |
| 75-84 | 1,491 (10.6%) | 2,056 (11.3%) | 1.03 (0.95, 1.12) | 0.98 (0.89, 1.07) | 0.602 |
| 85-94 (baseline) | 3,123 (22.1%) | 4,166 (23.0%) | 1.00 | 1.00 - | . |
| 95-104 | 3,493 (24.7%) | 4,232 (23.4%) | 0.91 (0.85, 0.97) | 0.97 (0.90, 1.04) | 0.363 |
| 105-114 | 2,808 (19.9%) | 3,235 (17.9%) | 0.86 (0.81, 0.92) | 0.94 (0.87, 1.01) | 0.097 |
| 115-124 | 1,576 (11.2%) | 1,939 (10.7%) | 0.92 (0.85, 1.00) | 1.05 (0.96, 1.14) | 0.313 |
| 125-134 | 754 (5.3%) | 1,040 (5.7%) | 1.03 (0.93, 1.15) | 1.11 (0.99, 1.24) | 0.075 |
| 135-144 | 344 (2.4%) | 495 (2.7%) | 1.08 (0.93, 1.25) | 1.13 (0.96, 1.32) | 0.136 |
| 145>= | 234 (1.7%) | 453 (2.5%) | 1.45 (1.23, 1.71) | 1.51 (1.26, 1.80) | <0.001 |

**Table 4.** Unadjusted and adjusted OR with 95% CI for the relationship between lowest SBP and hospital mortality (excluding those who died within the first 24 hours) when blood pressure was modelled as a categorical variable.

| **Lowest SBP** | **Survivors**  **n=14,123** | **Non-survivors**  **n=18,120** | **Unadjusted OR for mortality (95% CI)** | **Adjusted OR for mortality (95% CI)** | **p-value** |
| --- | --- | --- | --- | --- | --- |
| < 60 | 268 (1.9%) | 756 (4.2%) | 2.57 (2.23, 2.98) | 1.72 (1.47, 2.01) | <0.001 |
| 60-69 | 789 (5.6%) | 1,476 (8.1%) | 1.71 (1.55, 1.88) | 1.34 (1.20, 1.48) | <0.001 |
| 70-79 | 2,405 (17.0%) | 3,170 (17.5%) | 1.20 (1.12, 1.29) | 1.06 (0.98, 1.14) | 0.133 |
| 80-89 | 4,396 (31.1%) | 5,036 (27.8%) | 1.05 (0.99, 1.11) | 0.98 (0.92, 1.05) | 0.625 |
| 90-99 (baseline) | 3,871 (27.4%) | 4,241 (23.4%) | 1.00 | 1.00 | - |
| 100-109 | 1,624 (11.5%) | 2,119 (11.7%) | 1.19 (1.10, 1.29) | 1.19 (1.09, 1.29) | <0.001 |
| 110-119 | 506 (3.6%) | 779 (4.3%) | 1.41 (1.25, 1.58) | 1.34 (1.18, 1.53) | <0.001 |
| 120-129 | 163 (1.2%) | 348 (1.9%) | 1.95 (1.61, 2.36) | 1.98 (1.61, 2.44) | <0.001 |
| 130-139 | 61 (0.4%) | 115 (0.6%) | 1.72 (1.26, 2.35) | 1.62 (1.15, 2.27) | 0.006 |
| 140-149 | 21 (0.1%) | 42 (0.2%) | 1.83 (1.08, 3.09) | 1.72 (0.97, 3.06) | 0.064 |
| 150-159 | 12 (0.1%) | 25 (0.1%) | 1.90 (0.95, 3.79) | 1.71 (0.80, 3.68) | 0.167 |
| >/= 160 | 7 (0.0%) | 13 (0.1%) | 1.70 (0.68, 4.25) | 1.14 (0.42, 3.07) | 0.798 |

**Table 5.** Unadjusted and adjusted OR with 95% CI for the relationship between highest SBP and hospital mortality (excluding those who died within the first 24 hours) when blood pressure was modelled as a categorical variable.

*Categories where no OR for mortality is presented are those where there were either zero survivors, zero non-survivors or zero patients.*

| **Highest SBP** | **Survivors**  **n=14,123** | **Non-survivors**  **n=18,120** | **Unadjusted OR for mortality (95% CI)** | **Adjusted OR for mortality (95% CI)** | **p-value** |
| --- | --- | --- | --- | --- | --- |
| 60-69 | 0 (0.0%) | 1 (0.0%) | - | - | - |
| 70-79 | 1 (0.0%) | 3 (0.0%) | 1.96 (0.20, 19.07) | 2.19 (0.20, 23.45) | 0.516 |
| 80-89 | 5 (0.0%) | 19 (0.1%) | 2.49 (0.91, 6.81) | 2.40 (0.82, 7.01) | 0.110 |
| 90-99 | 27 (0.2%) | 52 (0.3%) | 1.26 (0.76, 2.10) | 1.22 (0.70, 2.11) | 0.479 |
| 100-109 (baseline) | 148 (1.0%) | 226 (1.2%) | 1.00 | 1.00 | - |
| 110-119 | 579 (4.1%) | 817 (4.5%) | 0.92 (0.73, 1.17) | 1.01 (0.78, 1.30) | 0.944 |
| 120-129 | 1,459 (10.3%) | 1,821 (10.0%) | 0.82 (0.66, 1.02) | 0.90 (0.71, 1.13) | 0.361 |
| 130-139 | 2,222 (15.7%) | 2,672 (14.7%) | 0.79 (0.64, 0.98) | 0.88 (0.70, 1.11) | 0.286 |
| 140-149 | 2,488 (17.6%) | 3,010 (16.6%) | 0.79 (0.64, 0.98) | 0.88 (0.70, 1.12) | 0.300 |
| 150-159 | 2,311 (16.4%) | 2,777 (15.3%) | 0.79 (0.64, 0.98) | 0.88 (0.70, 1.11) | 0.292 |
| >/= 160 | 4,883 (34.6%) | 6,722 (37.1%) | 0.90 (0.73, 1.11) | 0.97 (0.77, 1.21) | 0.765 |

**Table 6.** Unadjusted and adjusted OR with 95% CI for the relationship between lowest MAP and hospital mortality (including those who died within the first 24 hours) when blood pressure was modelled as a categorical variable.

*Categories where no OR for mortality is presented are those where there were either zero survivors, zero non-survivors or zero patients.*

| **Lowest MAP** | **Survivors**  **n=14,123** | **Non-survivors**  **n=24,336** | **Unadjusted OR for mortality (95% CI)** | **Adjusted OR for mortality (95% CI)** | **p-value** |
| --- | --- | --- | --- | --- | --- |
| <45 | 499 (3.5%) | 3,335 (13.7%) | 5.12 (4.63, 5.67) | 2.79 (2.50, 3.12) | <0.001 |
| 45-54 | 2,059 (14.6%) | 4,382 (18.0%) | 1.63 (1.53, 1.74) | 1.17 (1.09, 1.26) | <0.001 |
| 55-64 | 5,695 (40.3%) | 8,108 (33.3%) | 1.09 (1.04, 1.15) | 0.95 (0.90, 1.00) | 0.066 |
| 65-74 (baseline) | 4,582 (32.4%) | 5,977 (24.6%) | 1.00 | 1.00 | - |
| 75-84 | 1,001 (7.1%) | 1,865 (7.7%) | 1.43 (1.31, 1.56) | 1.51 (1.37, 1.66) | <0.001 |
| 85-94 | 223 (1.6%) | 466 (1.9%) | 1.60 (1.36, 1.89) | 1.81 (1.51, 2.17) | <0.001 |
| 95-104 | 46 (0.3%) | 124 (0.5%) | 2.07 (1.47, 2.90) | 2.20 (1.51, 3.21) | <0.001 |
| 105-114 | 15 (0.1%) | 53 (0.2%) | 2.71 (1.52, 4.81) | 2.43 (1.29, 4.57) | 0.006 |
| 115-124 | 2 (0.0%) | 19 (0.1%) | 7.28 (1.70, 31.28) | 6.35 (1.27, 31.67) | 0.024 |
| 125-134 | 1 (0.0%) | 3 (0.0%) | 2.30 (0.24, 22.12) | 1.96 (0.11, 33.97) | 0.644 |
| 134-145 | 0 (0.0%) | 4 (0.0%) | - | - | - |
| 145>= | 499 (3.5%) | 3,335 (13.7%) | - | - | - |

**Table 7.** Unadjusted and adjusted OR with 95% CI for the relationship between highest MAP and hospital mortality (including those who died within the first 24 hours) when blood pressure was modelled as a categorical variable.

*Categories where no OR for mortality is presented are those where there were either zero survivors, zero non-survivors or zero patients.*

| **Highest MAP** | **Survivors**  **n=14,123** | **Non-survivors**  **n=24,336** | **Unadjusted OR for mortality (95% CI)** | **Adjusted OR for mortality (95% CI)** | **p-value** |
| --- | --- | --- | --- | --- | --- |
| <45 | 0 (0.0%) | 65 (0.3%) | - | - | - |
| 45-54 | 1 (0.0%) | 137 (0.6%) | 80.27 (11.23, 574.04) | 42.91 (5.88, 313.17) | <0.001 |
| 55-64 | 22 (0.2%) | 366 (1.5%) | 9.75 (6.33, 15.03) | 5.47 (3.48, 8.60) | <0.001 |
| 65-74 | 277 (2.0%) | 1,219 (5.0%) | 2.58 (2.25, 2.96) | 1.80 (1.54, 2.10) | <0.001 |
| 75-84 | 1,491 (10.6%) | 3,212 (13.2%) | 1.26 (1.17, 1.36) | 1.13 (1.03, 1.22) | 0.006 |
| 85-94 (baseline) | 3,123 (22.1%) | 5,328 (21.9%) | 1.00 | 1.00 | - |
| 95-104 | 3,493 (24.7%) | 5,156 (21.2%) | 0.87 (0.81, 0.92) | 0.95 (0.88, 1.01) | 0.113 |
| 105-114 | 2,808 (19.9%) | 3,935 (16.2%) | 0.82 (0.77, 0.88) | 0.91 (0.85, 0.98) | 0.016 |
| 115-124 | 1,576 (11.2%) | 2,325 (9.6%) | 0.86 (0.80, 0.93) | 1.01 (0.93, 1.10) | 0.812 |
| 125-134 | 754 (5.3%) | 1,249 (5.1%) | 0.97 (0.88, 1.07) | 1.07 (0.96, 1.20) | 0.236 |
| 135-144 | 344 (2.4%) | 629 (2.6%) | 1.07 (0.93, 1.23) | 1.14 (0.98, 1.33) | 0.101 |
| 145>= | 234 (1.7%) | 715 (2.9%) | 1.79 (1.54, 2.09) | 1.85 (1.56, 2.19) | <0.001 |

**Table 8.** Unadjusted and adjusted OR with 95% CI for the relationship between lowest SBP and hospital mortality (including those who died within the first 24 hours) when blood pressure was modelled as a categorical variable.

| **Lowest SBP** | **Survivors**  **n=14,123** | **Non-survivors**  **n=24,336** | **Unadjusted OR for mortality (95% CI)** | **Adjusted OR for mortality (95% CI)** | **p-value** |
| --- | --- | --- | --- | --- | --- |
| < 60 | 268 (1.9%) | 2,651 (10.9%) | 7.86 (6.89, 8.98) | 4.04 (3.51, 4.66) | 0.000 |
| 60-69 | 789 (5.6%) | 2,483 (10.2%) | 2.50 (2.28, 2.74) | 1.71 (1.55, 1.89) | 0.000 |
| 70-79 | 2,405 (17.0%) | 4,280 (17.6%) | 1.41 (1.33, 1.51) | 1.16 (1.08, 1.25) | 0.000 |
| 80-89 | 4,396 (31.1%) | 5,981 (24.6%) | 1.08 (1.02, 1.15) | 0.99 (0.93, 1.06) | 0.876 |
| 90-99 (baseline) | 3,871 (27.4%) | 4,870 (20.0%) | 1.00 (., .) | 1.00 (., .) | . |
| 100-109 | 1,624 (11.5%) | 2,415 (9.9%) | 1.18 (1.10, 1.28) | 1.19 (1.09, 1.29) | 0.000 |
| 110-119 | 506 (3.6%) | 933 (3.8%) | 1.47 (1.30, 1.65) | 1.40 (1.23, 1.59) | 0.000 |
| 120-129 | 163 (1.2%) | 426 (1.8%) | 2.08 (1.73, 2.50) | 2.13 (1.73, 2.61) | 0.000 |
| 130-139 | 61 (0.4%) | 145 (0.6%) | 1.89 (1.40, 2.56) | 1.75 (1.25, 2.45) | 0.001 |
| 140-149 | 21 (0.1%) | 73 (0.3%) | 2.76 (1.70, 4.50) | 2.44 (1.41, 4.19) | 0.001 |
| 150-159 | 12 (0.1%) | 38 (0.2%) | 2.52 (1.31, 4.82) | 2.17 (1.05, 4.49) | 0.036 |
| >/= 160 | 7 (0.0%) | 41 (0.2%) | 4.66 (2.09, 10.39) | 3.17 (1.30, 7.76) | 0.011 |

**Table 9.** Unadjusted and adjusted OR with 95% CI for the relationship between highest SBP and hospital mortality (including those who died within the first 24 hours) when blood pressure was modelled as a categorical variable.

*Categories where no OR for mortality is presented are those where there were either zero survivors, zero non-survivors or zero patients.*

| **Highest SBP** | **Survivors**  **n=14,123** | **Non-survivors**  **n=24,336** | **Unadjusted OR for mortality (95% CI)** | **Adjusted OR for mortality (95% CI)** | **p-value** |
| --- | --- | --- | --- | --- | --- |
| < 60 | 0 (0.0%) | 39 (0.2%) | - | - | - |
| 60-69 | 0 (0.0%) | 53 (0.2%) | - | - | - |
| 70-79 | 1 (0.0%) | 101 (0.4%) | 17.75 (2.46, 128.24) | 12.39 (1.66, 92.26) | 0.014 |
| 80-89 | 5 (0.0%) | 249 (1.0%) | 8.75 (3.55, 21.58) | 6.68 (2.65, 16.84) | <0.001 |
| 90-99 | 27 (0.2%) | 415 (1.7%) | 2.70 (1.76, 4.14) | 2.42 (1.54, 3.81) | <0.001 |
| 100-109 (baseline) | 148 (1.0%) | 842 (3.5%) | 1.00 | 1.00 | - |
| 110-119 | 579 (4.1%) | 1,568 (6.4%) | 0.48 (0.39, 0.58) | 0.59 (0.47, 0.73) | <0.001 |
| 120-129 | 1,459 (10.3%) | 2,668 (11.0%) | 0.32 (0.27, 0.39) | 0.43 (0.35, 0.53) | <0.001 |
| 130-139 | 2,222 (15.7%) | 3,471 (14.3%) | 0.27 (0.23, 0.33) | 0.39 (0.32, 0.48) | <0.001 |
| 140-149 | 2,488 (17.6%) | 3,647 (15.0%) | 0.26 (0.21, 0.31) | 0.38 (0.31, 0.46) | <0.001 |
| 150-159 | 2,311 (16.4%) | 3,271 (13.4%) | 0.25 (0.21, 0.30) | 0.37 (0.31, 0.46) | <0.001 |
| >/= 160 | 4,883 (34.6%) | 8,012 (32.9%) | 0.29 (0.24, 0.34) | 0.41 (0.34, 0.50) | <0.001 |
